# The use of Qigong and Tai Chi in the management of temporomandibular joint dysfunction: A systematic review

**DOI:** 10.1002/hsr2.1639

**Published:** 2023-10-22

**Authors:** Afeez A. Salami, Kehinde K. Kanmodi, Dhanushka Leuke Bandara, Timothy O. Aladelusi, Jimoh Amzat, Dan Lin, Temitope O. Ojo, Ruwan D. Jayasinghe

**Affiliations:** ^1^ Faculty of Dentistry University of Puthisastra Phnom Penh Cambodia; ^2^ Department of Oral and Maxillofacial Surgery University College Hospital Ibadan Nigeria; ^3^ School of Dentistry University of Rwanda Kigali Rwanda; ^4^ School of Health and Life Sciences Teesside University Middlesbrough UK; ^5^ Department of Oral Medicine & Periodontology, Faculty of Dental Sciences University of Peradeniya Peradeniya Sri Lanka; ^6^ Department of Sociology Usmanu Danfodiyo University Sokoto Nigeria; ^7^ Department of Sociology University of Johannesburg Johannesburg South Africa

**Keywords:** disorder, dysfunction, Qigong, systematic review, Tai ji, temporomandibular joint

## Abstract

**Background and Aims:**

Qigong and Tai Chi (QTC) are mind‐body exercises involving a sequence of graceful movements, which promote self‐healing, meditation, and self‐cultivation. There is growing evidence that Qigong and/or Tai Chi therapy may improve the physical and psychological health among adults with multiple health conditions including joint movement. This systematic review aims to synthesize the available evidence on the type and effectiveness of QTC therapies used in the management of temporomandibular joint (TMJ) dysfunction.

**Methods:**

This systematic review followed the AMSTAR‐2 guideline, and it was reported based on guidelines in the PRISMA checklist. The review involved a systematic search of nine electronic databases. After deduplication and screening of the literature retrieved from the search, only one article was included based on the review's inclusion criteria. Data was extracted from this article and synthesized.

**Results:**

The only included article was on a non‐randomized control trial which investigated the role of Tai Chi Qigong (a form of Qigong) therapy in improving joint mobility and alleviating trismus among 52 survivors of nasopharyngeal carcinoma who had TMJ disorders. The study reported, at different time intervals (at pretest; at mid‐intervention; at posttest; and at follow‐up posttest), that no significant difference (*p* > 0.05) was observed concerning mouth opening capacity between the intervention group and the control group. However, over time, less severe deterioration in mouth‐opening was noted among the participants in the intervention group (*p* = 0.181) as compared to the control group (*p* < 0.001).

**Conclusion:**

The role of QTC therapies in improving joint mobility and alleviating trismus is not yet fully understood, but it seems promising. The available evidence is inadequate to categorically conclude on the efficacy of these therapies. Further studies will be required to fully assess the effectiveness of QTC therapies in the management of TMJ dysfunction.

## 1 INTRODUCTION

The temporomandibular joint (TMJ) is a complex diarthrodial joint that is formed by the articulation of the squamous part of temporal bone and the mandibular condyle and it is interposed by a fibrocartilaginous TMJ disc.^1^ The TMJ is unique structure in the articulatory system which is functionally adapted to perform a wide range of movements.^2^ However, changes in the movement of the joint can occur as a result of conditions such as joint dysfunction, degenerative changes, inflammation, infective process, neoplasm, trauma, stress, and muscle tension, which could manifest as jaw pain, limitation in jaw movement, stiffness of the jaw, and clicking or popping in the joint.^1^, ^3^, ^4^, ^5^ These clinical characteristics are associated with reduced quality of life.^6^

Qigong and Tai Chi (QTC) are forms of mind‐body exercises that have been used since their origin in ancient China, which promote self‐healing, meditation, and self‐cultivation. Based on concepts in Traditional Chinese Medicine (TCM), these practices comprise coordinated body posture and movements, meditation, deep rhythmic breathing, and mental focus.^7^, ^8^ Tai Chi consists of a sequence of graceful movements coupled with deep and slow diaphragmatic breathing. Similarly, the Qigong exercise consists of a series of breath practices involved with body movement and meditation to attain deep focus and a relaxed state. In TCM, poor health is attributable to the stagnated “Qi,” the vital energy, within the body. Thus, rhythmic movements improve the flow of Qi in the body and resolve any obstruction that might have resulted in stagnant Qi.^9^ To move Qi within the body, practitioners use intent or imaginary training to drive the flow of Qi in the paths of the meridians described by TCM concepts.^10^ Therefore, it is considered that based on these concepts, QTC could promote physical and mental well‐being.^9^, ^11^

There is growing evidence that QTC may improve the physical and psychological health among adults with multiple health conditions including neurological disorders, metabolic diseases, cancers, musculoskeletal diseases, cardiovascular diseases, and cognitive‐psychological disorders.^12^, ^13^ Thus, the popularity of QTC among all ages is gaining worldwide attention.^14^

Furthermore, the calm and low‐impact forms of coordinated physical exertion that are produced in QTC may alleviate stress and tension in the muscles of the face and neck.^15^, ^16^ This is further supported by evidence that practicing QTC can improve the range of joint motion and reduce pain and muscle tension in individuals.^7^, ^17^ Additionally, these practices' mindfulness and relaxation components may help individuals cope with stress and anxiety, which can trigger or worsen impaired joint function.^18^ However, no known study has conducted a systematic review to synthesize the existing research evidence on the effects of Qigong and/or Tai Chi in the management of TMJ dysfunction; hence, this systematic review was conducted.

With the growing interest in the use of adjuvant nonpharmacological therapies in oral and maxillofacial care,^19^, ^20^, ^21^ the need for evidence synthesis on the applications of Qigong and/or Tai Chi in the management of TMJ dysfunction, through systematic review approach, cannot be overemphasized as the outcomes from such review will provide the highest level of reliable clinical evidence that can be used in making evidence‐based clinical decisions in patient care.^22^, ^23^ Importantly, this review will provide insights into whether Qigong and/or Tai Chi can be used solely, or as an adjuvant, in the management of TMJ problems or perhaps avoided in the management of TMJ problems.

## 2 MATERIALS AND METHODS

### 2.1 Title and protocol registration

The title and protocol of this systematic review was registered with PROSPERO (International Prospective Register of Systematic Reviews), and its registration number was CRD42023417665.

### 2.2 Research design

The methodology of this systematic review was reported based on guidelines in the Preferred Reporting Items for Systematic Reviews and Meta‐analysis checklist.^22^, ^24^ The AMSTAR 2 checklist also informed the conduct of the review's methodological processes.^25^

### 2.3 Research question

This systematic review seeks to answer these research questions:

1. What are the types of Qigong therapy used in the management of TMJ dysfunction?
2. How effective is Qigong therapy in the management of TMJ dysfunction?
3. What are the types of Tai Chi therapy used in the management of TMJ dysfunction?
4. How effective is Tai Chi therapy in the management of TMJ dysfunction?

### 2.4 Literature search strategy

To answer the above review questions, we identified and retrieved relevant literature by conducting a systematic search of nine databases which included: PubMed, SCOPUS, AMED, CINAHL Ultimate, Dentistry and Oral Sciences Source, SPORTDiscus with Full text, APA PsycInfo, APA PsycArticles, and Psychology and Behavioral Sciences Collection. The search was conducted on April 18, 2023, to retrieve all relevant literature published from inception till the search date, with the aid of “OR” and “AND” Boolean operators, using different search terms on Tai Chi, Qigong, and TMJ. The search terms used were obtained from the Medical subject Heading (MeSH) dictionary, Thesaurus, and search strings obtained from existing structured reviews on TMJ, Tai Chi, and Qigong.^26^, ^27^, ^28^, ^29^, ^30^ Supporting Information: Tables S1 to S3 (Appendix) depict the search strings used for the literature search strategy.

### 2.5 Study selection

All retrieved articles were imported into the Rayyan web application for deduplication and the deduplicated copies were screened for inclusion into this systematic review using comprehensive eligibility criteria for the inclusion of articles.^31^

The inclusion criteria include:

- Publications that were peer‐reviewed journal articles.
- Articles that were published in English or Chinese.
- Articles reporting empirical research findings, of any research design, on the effects of Qigong and/or Tai Chi therapy on TMJ functioning.
- Literatures with accessible full texts.

However, those publications that failed to meet the above criteria were excluded from the systematic review. These include:

- Articles that were published in non‐peer‐reviewed journals.
- Peer‐reviewed journal articles that did not report empirical data (e.g., reviews, editorials, commentaries etc.) on the effects of Qigong and/or Tai Chi therapy on TMJ functioning.
- Articles that were not published in English or Chinese.
- Articles reporting empirical research findings on the effects of other treatment modalities, other than Qigong and/or Tai Chi therapy, on TMJ functioning.
- Articles reporting empirical research findings on the effects of Qigong and/or Tai Chi therapy on the functioning of other joints.
- Articles without accessible full texts (in this context, an article with inaccessible full text was considered a non‐open access article whose full text could not be provided within 4 weeks after its request from the corresponding author or the British Inter‐Library Loan).

By adhering strictly to these eligibility criteria, a two‐stage screening process was carried out by two reviewers (K. K. K. and A. A. S.). In the first stage, titles and abstracts screening were done to exclude all irrelevant articles while in the second stage, full‐text screening of all articles that passed the first stage of screening was done for the possibility of inclusion. Notably, conflicts that arose in the process of inclusion/exclusion of any literature were resolved through critical discussions between both reviewers and other senior members of the research team (D. L. B. and R. D. J.). Only those articles that met all the inclusion criteria were finally considered eligible for inclusion into the systematic review (Supporting Information: Table S4).

### 2.6 Risk of bias assessment

The risk of bias assessment was conducted using the Mixed Methods Appraisal version 2018 Tool^32^ (Table 1) by two reviewers (K. K. K. and A. A. S.). Notably, the Mixed Methods Appraisal version 2018 Tool uses a scale of 0−7 from a set of seven questions to assess the quality of the included articles. In this tool, the first two questions are general questions applicable to all study designs and the remaining five questions are study‐design specific questions which include qualitative study design, quantitative randomized control trials, quantitative non‐randomized control trials, quantitative descriptive study, and the mixed methods design. The grading of the studies was conducted according to approach adopted from Clark et al.^33^ where a score of 1 was given to a response of “Yes” to the appraisal question, 0.5 score given to a response of “I cannot tell,” and score of 0 given to a response of “No” to the appraisal question. The appraisal questions were answered for each article and scored appropriately to determine the quality of such article.

**Table 1:** Risk of bias assessment outcome of the included article (*n* = 1).

No	Author(s) (year)	Study design	MMAT version 2018 questions (Hong et al.^32^)							Total score (over 7)	Grading	Status
			Screening questions		Questions specific to study design							
			S1	S2	1st	2nd	3rd	4th	5th			
1.	Fong et al.^34^	Non‐randomized trial	0.5	0.5	0.5	1.0	1.0	0	1.0	4.5	Above average quality	MS

*Note*: Yes, 1.0 point; I can't tell, 0.5 point; No, 0 point; MS, methodologically sound.

A cumulative score range of 4−7 points was rated as above average quality, a cumulative score of 3.5 points was rated as average quality, and a score range of 1−3 points was rated as below average quality for each of the articles assessed. The article with cumulative score of 4−7 points were considered methodologically sound.

### 2.7 Data extraction

A bespoke data extraction sheet, developed based on the Joanna Briggs Institute's guidelines for data extraction in systematic reviews,^35^ was used to extract the following data from the included literature: author names, publication year, study design, study objectives, study population attributes including sociodemographic data of participants, study instruments, study results, and conclusions (Supporting Information: Table S5).

### 2.8 Data synthesis

Narrative synthesis approach was used to synthesize the data extracted from the included literature. Meta‐analysis could not be done due to lack of adequate number of literatures (a minimum of two literatures) needed to accomplish such analysis.^35^

## 3 RESULTS

### 3.1 Literature search outcome

**Figure 1:**
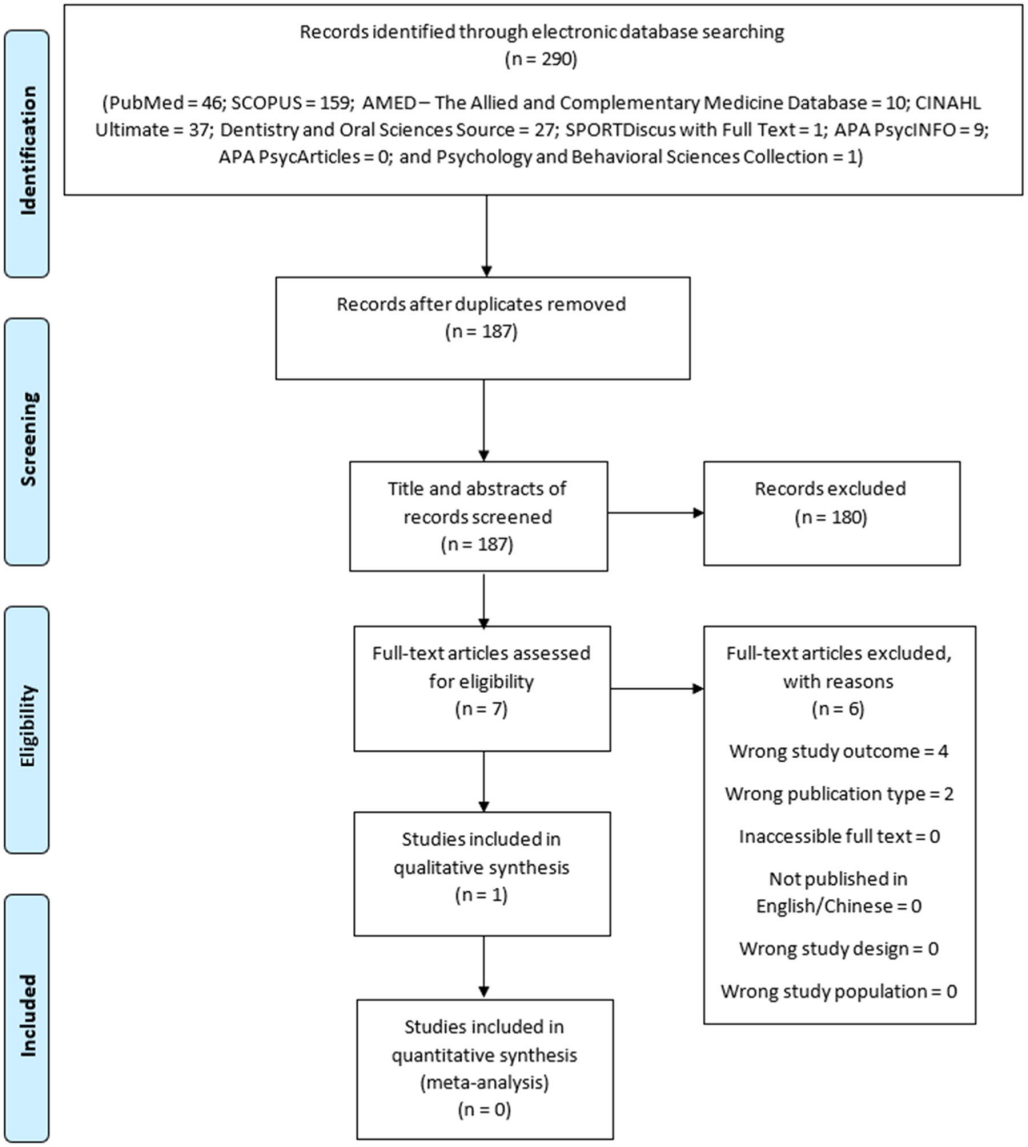
Flow chart diagram.

A total of 290 publications were retrieved from the database search (PubMed = 46; SCOPUS = 159; AMED [The Allied and Complementary Medicine Database] = 10; CINAHL Ultimate = 37; Dentistry and Oral Sciences Source = 27; SPORTDiscus with Full Text = 1; APA PsycINFO = 9; APA PsycArticles = 0; and Psychology and Behavioral Sciences Collection = 1) (Figure 1).

### 3.2 Outcome of literature deduplication

Out of the 290 publications retrieved, 103 publications were duplicates and were deleted. The remaining 187 publications were single entry records and were thereafter subjected to screening.

### 3.3 Literature screening outcome

The titles and abstracts of the deduplicated 187 publications were screened for relevance, out of which only seven articles were considered for full text screening. Out of these seven articles (Supporting Information: Table S4), only one article, by Fong et al.,^34^ met all the inclusion criteria and, hence, was considered for risk of bias assessment.

### 3.4 Risk of bias assessment outcome

The included article by Fong et al.^34^ was considered for risk of bias assessment using the Mixed Methods Assessment version 2018 Tool after meeting all the inclusion criteria. After a thorough assessment of this study, we considered this study to be methodologically sound (Cumulative score of 4.5) as depicted in Table 1 below.

During this assessment, we were unsure if the authors had a clear research question while going into this research even when the aims were stated. Therefore, we could not conclude if the collected data has addressed their research questions. Similarly, we could not tell if the participants were representative of the target population because over 40% of the test group dropped out of the study which could have significantly affected the outcome.

Furthermore, cofounders were not properly accounted for in this study. First, due to its unrandomized nature and second, the fact that both groups were still receiving hospital care which may not be identical during the study period.

Summarily, this study is of above average quality (cumulative score of 4.5) and can be considered as methodologically sound.

### 3.5 Study design

The included article was a report of a study which adopted a single‐blinded, non‐randomized, controlled clinical trial design.^34^

### 3.6 Study location

The study location was not clearly defined in the included article.^34^

### 3.7 Study population

The study population in the included article were 52 survivors of nasopharyngeal carcinoma who had TMJ disorders.^34^

### 3.8 Intervention

The intervention delivered in the study was a training on Tai Chi Qigong: a modified Qigong. However, the specific Qigong training type delivered to the intervention group was not clearly defined in the study.^34^ Only the intervention group (*n* = 25) received the Tai Chi Qigong intervention while the control group (*n* = 25) did not receive it. The intervention was a 1.5 h weekly intervention which spanned over a period of 6 consecutive months.^34^

### 3.9 Study instrument

The study instrument used to collect data from the participants concerning the intervention and TMJ functional outcomes were questionnaire and a plastic ruler marked in millimeters.^34^ The plastic ruler was used to obtain measurement of the inter‐incisal distance to access maximum unassisted mouth opening capacity of the study participants.^34^

### 3.10 Outcome measurement

Measurements on the maximum unassisted mouth opening capacity of the study participants were obtained at intervals: 1 week before the commencement of the Tai Chi Qigong training intervention (pretest), at mid‐intervention (3 months after the commencement of Tai Chi Qigong training intervention), within 1‐week post‐completion of the Tai Chi Qigong training intervention (posttest), and 6 months after the completion of Tai Chi Qigong training intervention (follow‐up posttest).^34^

### 3.11 Effects of Tai Chi Qigong on TMJ

At baseline, the mean (±standard deviation) inter‐incisal distance at maximum unassisted mouth opening capacity was 3.42 cm (±1.08 cm) in the intervention group and 4.52 cm (±1.43 cm) in the control group.^34^ There was no significant difference (*p* > 0.05) reported on the mouth opening between the groups at different time intervals: at pre‐test; at mid‐intervention; at posttest; and at follow‐up posttest.^34^ However, over time, less severe deterioration in mouth‐opening was noted among the participants in the intervention group (*p* = 0.181) as compared to the control group (*p* < 0.001).^34^

## 4 DISCUSSION

The TMJ produces functional movement at the articulation between the mandible and the maxilla. These functional movements are highly critical in maintaining oral function.^36^, ^37^ It is also distinct from other joints due to its anatomical arrangement, the presence of a rigid endpoint and the need for simultaneous movements of both components to create smooth movements.^36^ This unique structure also makes the TMJ vulnerable to functional impairments due to the diseases of the joint itself or masticatory muscle disorders.^38^

There are multiple factors (such as degenerative changes, inflammation, infective process, neoplasm, trauma, stress, and muscle tension) responsible for TMJ's functional impairments.^1^, ^3^, ^4^, ^5^ Trismus is one of the clinical features of TMJ disorders, and this could be aggravated by a variety of factors such as radiotherapy, reduced nutrition, and compromised oral health.^37^, ^38^, ^39^, ^40^ Other clinical features of TMJ disorder include clicking and/or crepitus in the joint during function, pain, and joint immobility.^40^ In assessing the outcome of any therapeutic approach in TMJ care, a subjective assessment of the joint should be carried out to ensure reproducibility.^38^ In most clinical situations, assessment is done using multiple clinical parameters (such as joint movements/stiffness, tenderness, and crepitations) as they provide simple and accurate measurements.^37^, ^38^ The assessment of TMJ movement ideally should involve a wide range of dimensions covering different joint movements such as mouth opening, lateral movements, and anterior movements.^38^ Mouth opening capacity, which is measured by the inter‐incisal opening, could be done in different stages (assisted, non‐assisted, and pain‐free stages).^38^ In the study analyzed in this systematic review,^34^ unassisted mouth opening capacity was the clinical parameter used.

QTC therapies has been utilized in clinical practice for the management of diverse body joint disorders.^41^, ^42^, ^43^, ^44^, ^45^, ^46^, ^47^, ^48^, ^49^, ^50^, ^51^, ^52^ For example, Tai Chi has been used to reduce pain and improve physical and psychological well‐being in patients with rheumatoid arthritis, fibromyalgia, neurological, and cardiovascular diseases.^41^, ^42^, ^43^, ^44^, ^45^ Also, Tai Chi confers significant clinical improvements in muscle strength, joint flexibility, physical and mental functions, endurance, and general quality of life of patients with knee osteoarthritis.^46^, ^47^ Also, Qigong therapy has been found to be effective in ensuring postural stability, improved knee proprioception and muscle strength, pain alleviation, and improved joint mobility in elderly patients with symptoms of knee joint osteoarthritis.^48^, ^49^, ^50^, ^51^, ^52^ However, based on the synthesized evidence obtained in this review, it can be inferred that the effectiveness of QTC in improving TMJ pain and mobility, alleviating trismus, and other features of TMJ disorders is still inconclusive, as the synthesis was based on only one empirical study, and no significant improvement in unassisted mouth opening capacity was recorded, after repeated measurements, among recipients of QTC therapies.

Furthermore, in the study evaluated in this systematic review,^34^ the exact regimen of the Tai Chi Qigong (a form of Qigong) therapy was not clearly defined, thus compromising the reproducibility of the study. Hence, further studies with better‐streamlined study designs would be needed to further assess the effect of QTC on the TMJ.

This systematic review has its limitations. The review's synthesis was based on the findings of just one study. Also, the only included study was carried out on a small sample of patients, and it assessed only one parameter (i.e., mouth‐opening capacity) to evaluate TMJ function; hence, the synthesized evidence in the review should be interpreted with strict caution. However, despite the above‐mentioned limitations, a clear search strategy which involved a very wide range of databases followed by strict selection criteria in the interest of obtaining high quality evidence could be highlighted as a strength of this review. Due to the thoroughness of this systematic review, it can be categorically asserted that this review has provided some evidence on the status of empirical research on the use of QTC therapies in the management of TMJ dysfunction. Also, the lack of robust quantity of articles in this review has provided a clear direction for future original research especially in providing cost‐effective alternative treatment for patients with TMJ dysfunction through the use of QTC therapies.

## 5 CONCLUSION

The role of QTC therapies in improving joint mobility and alleviating trismus is not yet fully understood, but it seems promising. The available evidence is inadequate to categorically conclude on the efficacy of this therapy. Further studies will be required to fully assess the effectiveness of QTC therapies in the management of TMJ dysfunction.

## AUTHOR CONTRIBUTIONS

**Afeez A. Salami**: Data curation; formal analysis; investigation; methodology; resources; software; validation; visualization; writing—original draft; writing—review and editing. **Kehinde K. Kanmodi**: Conceptualization; data curation; formal analysis; funding acquisition; investigation; methodology; project administration; resources; software; supervision; validation; visualization; writing—original draft; writing—review and editing. **Dhanushka Leuke Bandara**: Data curation; formal analysis; investigation; methodology; resources; writing—original draft; writing—review and editing. **Timothy O. Aladelusi**: Resources; writing—original draft. **Jimoh Amzat**: Resources; writing—original draft; writing—review and editing. **Dan Lin**: Investigation; writing—review and editing. **Temitope O. Ojo**: Investigation; resources. **Ruwan D. Jayasinghe**: Formal analysis; investigation; methodology; project administration; resources; supervision; validation; visualization; writing—original draft; writing—review and editing. All authors have read and approved the final version of the manuscript.

## CONFLICT OF INTEREST STATEMENT

Kehinde Kazeem Kanmodi is an Editorial Board member of Health Science Reports and a coauthor of this article. To minimize bias, they were excluded from all editorial decision‐making related to the acceptance of this article for publication. The remaining authors declare no conflict of interest.

## ETHICS STATEMENT

This study did not collect data from human or animal subjects but an open research repository.

## TRANSPARENCEY STATEMENT

The lead author, Afeez Abolarinwa Salami, affirms that this manuscript is an honest, accurate, and transparent account of the study being reported; that no important aspects of the study have been omitted; and that any discrepancies from the study as planned (and, if relevant, registered) have been explained.

## Supporting information

Supporting information.

## Data Availability

Data sharing is not applicable to this article as no new data were created or analyzed in this study. The lead author, Afeez Abolarinwa Salami, had full access to all of the data in this study and takes complete responsibility for the integrity of the data and the accuracy of the data analysis.
