# Supplementary material for: The use of Qigong and Tai Chi in the management of temporomandibular joint dysfunction: A systematic review
Source: Health Sci Rep. 2023 Oct 22;6(10):e1639. doi: 10.1002/hsr2.1639 (PMC10591026; doi:10.1002/hsr2.1639)
Supplement: Supplementary file 1 — Supporting information. [file HSR2-6-e1639-s001.docx]

**SUPPLEMENTARY FILE**

**Table S1. Search string for PubMed database search**

| **Tag** | **Subject search** | **Search String** |
| --- | --- | --- |
| #1 | Qigong/Tai chi | ((((((((((((((((((((((((((Qigong[Title/Abstract]) OR (qi gong[Title/Abstract])) OR (chi kung[Title/Abstract])) OR (chi 'ung[Title/Abstract])) OR (chi gung[Title/Abstract])) OR (Tao yin[Title/Abstract])) OR (Wu Qin Xi[Title/Abstract])) OR (wu xin xi[Title/Abstract])) OR (Ba Duan Jin[Title/Abstract])) OR (Yi Jin Jing[Title/Abstract])) OR (Liu Zi Jue[Title/Abstract])) OR (Shi Er Duan Jin[Title/Abstract])) OR (Ma Wang Dui Dao Yin[Title/Abstract])) OR (complementary medic*[Title/Abstract])) OR (traditional chinese medic*[Title/Abstract])) OR (alternative medic*[Title/Abstract])) OR (complementary therapy[Title/Abstract])) OR (traditional chinese therapy[Title/Abstract])) OR (alternative therapy[Title/Abstract])) OR (Tai chi[Title/Abstract])) OR (Tai ji[Title/Abstract])) OR (tai jin[Title/Abstract])) OR (taiji[Title/Abstract])) OR (taichi[Title/Abstract])) OR (Tai chi chuan[Title/Abstract])) OR (Taichiquan[Title/Abstract])) OR (Tai chi Quan[Title/Abstract]) |
| #2 | Temporomandibular | ((((((temporomandibular[Title/Abstract]) OR (TMJ[Title/Abstract])) OR (TMD[Title/Abstract])) OR (Costen[Title/Abstract])) OR (craniomandibular[Title/Abstract])) OR (giglimosphenoidal[Title/Abstract])) OR (bruxism[Title/Abstract]) |
| #3 | #2 AND #3 | (#2) AND (#3) |

**Table S2. Search string for SCOPUS database search**

| **Tag** | **Subject search** | **Search String** |
| --- | --- | --- |
| #1 | Qigong/Tai chi | ( TITLE-ABS-KEY ( qigong )  OR  TITLE-ABS-KEY ( "qi gong" )  OR  TITLE-ABS-KEY ( "chi kung" )  OR  TITLE-ABS-KEY ( "chi 'ung" )  OR  TITLE-ABS-KEY ( "chi gung" )  OR  TITLE-ABS-KEY ( "Tao yin" )  OR  TITLE-ABS-KEY ( "Wu Qin Xi" )  OR  TITLE-ABS-KEY ( "wu xin xi" )  OR  TITLE-ABS-KEY ( "Ba Duan Jin" )  OR  TITLE-ABS-KEY ( "Yi Jin Jing" )  OR  TITLE-ABS-KEY ( "Liu Zi Jue" )  OR  TITLE-ABS-KEY ( "Shi Er Duan Jin" )  OR  TITLE-ABS-KEY ( "Ma Wang Dui Dao Yin" )  OR  TITLE-ABS-KEY ( "complementary medic*" )  OR  TITLE-ABS-KEY ( "traditional chinese medic*" )  OR  TITLE-ABS-KEY ( "alternative medic*" )  OR  TITLE-ABS-KEY ( "complementary therapy" )  OR  TITLE-ABS-KEY ( "traditional chinese therapy" )  OR  TITLE-ABS-KEY ( "alternative therapy" )  OR  TITLE-ABS-KEY ( "Tai chi" )  OR  TITLE-ABS-KEY ( "Tai ji" )  OR  TITLE-ABS-KEY ( "tai jin" )  OR  TITLE-ABS-KEY ( taiji )  OR  TITLE-ABS-KEY ( taichi )  OR  TITLE-ABS-KEY ( "Tai chi chuan" )  OR  TITLE-ABS-KEY ( taichiquan )  OR  TITLE-ABS-KEY ( "Tai chi Quan" ) ) |
| #2 | Temporomandibular | ( TITLE-ABS-KEY ( temporomandibular )  OR  TITLE-ABS-KEY ( tmj )  OR  TITLE-ABS-KEY ( tmd )  OR  TITLE-ABS-KEY ( costen )  OR  TITLE-ABS-KEY ( craniomandibular )  OR  TITLE-ABS-KEY ( giglimosphenoidal )  OR  TITLE-ABS-KEY ( bruxism ) ) |
| #3 | #2 AND #3 | (#2) AND (#3) |

**Table S3. Search string for other database (AMED – The Allied and Complementary Medicine Database; CINAHL Ultimate; Dentistry and Oral Sciences Source; SPORTDiscus with Full Text; APA PsycInfo; APA PsycArticles; and Psychology and Behavioural Sciences Collection) search via EBSCO*Host* interface**

| **Tag** | **Subject search** | **Search String** |
| --- | --- | --- |
| S1 | Qigong/Tai chi | AB Qigong OR AB qi gong OR AB chi kung OR AB chi 'ung OR AB chi gung OR AB Tao yin OR AB Wu Qin Xi OR AB wu xin xi OR AB Ba Duan Jin OR AB Yi Jin Jing OR AB Liu Zi Jue OR AB Shi Er Duan Jin OR AB Ma Wang Dui Dao Yin OR AB complementary medic* OR AB traditional chinese medic* OR AB alternative medic* OR AB complementary therapy OR AB traditional chinese therapy OR AB alternative therapy OR AB Tai chi OR AB Tai ji OR AB tai jin OR AB taiji OR AB taichi OR AB Tai chi chuan OR AB Taichiquan OR AB Tai chi Quan |
| S2 | Temporomandibular | AB Temporomandibular OR AB TMJ OR AB TMD OR AB Costen OR AB craniomandibular OR AB giglimosphenoidal OR AB bruxism |
| S3 | S2 AND S3 | S2 AND S3 |

**Table S4. List of literatures considered for full text screening and their screening outcomes.**

| **Item** | **Citation** | **Included** | **Excluded (Reasons)** |
| --- | --- | --- | --- |
| 1 | Ritenbaugh C, Hammerschlag R, Dworkin SF, Aickin MG, Mist SD, Elder CR, Harris RE. Comparative effectiveness of traditional Chinese medicine and psychosocial care in the treatment of temporomandibular disorders-associated chronic facial pain. J Pain. 2012 Nov;13(11):1075-89. doi: 10.1016/j.jpain.2012.08.002. Epub 2012 Oct 9. PMID: 23059454; PMCID: PMC3490702. |  | Yes (Wrong study outcome) |
| 2 | Fong SS, Ng SS, Lee HW, Pang MY, Luk WS, Chung JW, Wong JY, Masters RS. The effects of a 6-month Tai Chi Qigong training program on temporomandibular, cervical, and shoulder joint mobility and sleep problems in nasopharyngeal cancer survivors. Integr Cancer Ther. 2015 Jan;14(1):16-25. doi: 10.1177/1534735414556508. Epub 2014 Nov 18. PMID: 25411207. | Yes |  |
| 3 | Ritenbaugh C, Hammerschlag R, Calabrese C, Mist S, Aickin M, Sutherland E, Leben J, Debar L, Elder C, Dworkin SF. A pilot whole systems clinical trial of traditional Chinese medicine and naturopathic medicine for the treatment of temporomandibular disorders. J Altern Complement Med. 2008 Jun;14(5):475-87. doi: 10.1089/acm.2007.0738. PMID: 18564953; PMCID: PMC2756304. |  | Yes (Wrong study outcome) |
| 4 | Li GH. [Treatment of temporomandibular joint disorder by traditional Chinese medicine]. Zhong Xi Yi Jie He Xue Bao. 2003 Nov;1(4):258, 276. Chinese. doi: 10.3736/jcim20030407. PMID: 15339524. |  | Yes (Wrong publication type) |
| 5 | Ryan M.K. Temporomandibular dysfunction: traditional Chinese medicine approach. J Bodyw Mov Ther. 1997;1(4): 213-214 |  | Yes (Wrong publication type) |
| 6 | Yamaner FE, Celakil T, Gökcen Roehlig B. Comparison of the efficiency of two alternative therapies for the management of temporomandibular disorders. Cranio. 2022 May;40(3):189-198. doi: 10.1080/08869634.2020.1727667. Epub 2020 Feb 15. PMID: 32065060. |  | Yes (Wrong study outcome) |
| 7 | Raphael KG, Klausner JJ, Nayak S, Marbach JJ. Complementary and alternative therapy use by patients with myofascial temporomandibular disorders. J Orofac Pain. 2003 Winter;17(1):36-41. PMID: 12756929. |  | Yes (Wrong study outcome) |

**Table S5. Data extraction sheet**

| **Author (Year)** | **Study design** | **Purpose of study** | **Sample size** | **Participants’ characteristics** | **Intervention** | **Study instruments** | **Results** | **Conclusions** |
| --- | --- | --- | --- | --- | --- | --- | --- | --- |
| Fong et al. (2015) | Single-blinded, non-randomized, controlled clinical trial design | To investigate the efficacy of Tai Chi Qigong in the optimization of temporomandibular joint (TMJ), cervical, and shoulder joint mobility and reducing sleep problems in nasopharyngeal carcinoma survivors. | 52 | Survivors of nasopharyngeal carcinoma who had TMJ disorders | Tai Chi Qigong | A questionnaire and a plastic ruler marked in millimetres. The plastic ruler was used to obtain measurement of the inter-incisal distance to access maximum unassisted mouth opening capacity of the study participants | At baseline, the mean (± standard deviation) inter-incisal distance at maximum unassisted mouth opening capacity was 3.42cm (±1.08cm) in the intervention group and 4.52cm (±1.43cm) in the control group. There was no significant difference (P > 0.05) reported on the mouth opening between the groups at different time intervals: at pre-test; at mid-intervention; at post-test; and at follow-up post-test. However, over time, less severe deterioration in mouth-opening was noted among the participants in the intervention group (P = 0.181) as compared to the control group (P < 0.001). | Tai Chi Qigong therapy maintained TMJ mobility among the intervention group. |
